# Supplementary material for: Oxepinamide F biosynthesis involves enzymatic d-aminoacyl epimerization, 3H-oxepin formation, and hydroxylation induced double bond migration
Source: Nat Commun. 2020 Oct 1;11:4914. doi: 10.1038/s41467-020-18713-0 (PMC7530659; doi:10.1038/s41467-020-18713-0)
Supplement: Supplementary file 1 — supplementary Information [file 41467_2020_18713_MOESM1_ESM.pdf]

Supporting Information

Oxepinamide F biosynthesis involves enzymatic D-aminoacyl epimerization, 3*H*-oxepin formation, and hydroxylation induced double bond migration

Zheng et al.

This PDF file contains:

- Supplementary Tables
- Supplementary Figures
- Supplementary Note 1
- Supplementary References

Supplementary Tables

Supplementary Table 1. Putative functions of the genes from *opa* gene cluster

Protein	No.	Size (aa)	coverage/identity, homologous protein, organism	Putative function
OpaA	KIA75458	3901	95/39 nonribosomal peptide synthetase FmqA, Q4WLW5.1, *Aspergillus fumigatus Af293*	quinazolinone formation
OpaB	KIA75457	575	84/30, Cytochrome P450 PenB, ANY57880.1, *Penicillium thymicola*	P450, Oxepin formation
OpaC	KIA75456	462	93/40, FAD-dependent monooxygenase NodY2, A0A2I6PIZ8.1, *Hypoxylon pulicicidum*	C-12 hydroxylation
OpaD	KIA75455	587	98/51, efflux pump RoqT, KAF3399802.1, *Penicillium rolfsii*	transport
OpaE	KIA75454	274	79/28 hydantoin racemase, Q9F466.1, *Paenarthrobacter aurescens*	C-15 epimerization
OpaF	KIA75453	420	86/39, *O*-methyltransferase KntB, A2QK65.1 (XP_001402308.1), *Aspergillus niger CBS 513.88*	*O*-methylation

Supplementary Table 2. Strains used in this study

Stains	Genotype
Wild type	*A. ustus* 3.3904
ΔopaA	*ΔopaA*::*hph* in *A. ustus* 3.3904
ΔopaB	*ΔopaB*::*hph* in *A. ustus* 3.3904
ΔopaC	*ΔopaC*::*hph* in *A. ustus* 3.3904
ΔopaE	*ΔopaE*::*hph* in *A. ustus* 3.3904
ΔopaF	*ΔopaF*::*hph* in *A. ustus* 3.3904
A.nidulans LO8030	*pyroA4*, *riboB2*, *pyrG89*, *nkuA::argB*
	sterigmatocystin cluster (*AN7804-AN7825*)Δ,
	emericellamide cluster (*AN2545-AN2549*)Δ,
	asperfuranone cluster (*AN1039-AN1029*)Δ,
	monodictyphenone cluster (*AN10023-AN10021*)Δ,
	terrequinone cluster (*AN8512-AN8520*)Δ,
	austinol cluster part 1 (*AN8379-AN8384*)Δ,
	austinol cluster part 2 (*AN9246-AN9259*)Δ,
	F9775 cluster (*AN7906-AN7915*)Δ,
	asperthecin cluster (*AN6000-AN6002*)Δ
LZ61	*gpdA::opaB::AfpyrG* in *A. nidulans* LO8030

Supplementary Table 3. Plasmids constructed and used in this study

Plasmids	Description
p5HY	Two-third of the hph resistance gene at the 5´-end, originated from the pUChph and inserted into pESC-URA. For gene replacement using hph as selection marker.
p3YG	Two-third of the *hph* resistance gene at the 3´-end, originated from the pUChph and inserted into pESC-URA. For gene replacement using *hph* as selection marker.
pLZ131 (p5HY-*opaA*)	a 1453 bp US PCR fragment of *opaA* from genomic DNA of *A. ustus* 3.3904 inserted in p5HY.
pLZ132 (p3YG-*opaA*)	a 1427 bp DS PCR fragment of *opaA* from genomic DNA of *A. ustus* 3.3904 inserted in p3YG.
pLZ133 (p5HY-*opaB*)	a 1252 bp US PCR fragment of *opaB* from genomic DNA of *A. ustus* 3.3904 inserted in p5HY.
pLZ134 (p3YG-*opaB*)	a 1285 bp DS PCR fragment of *opaB* from genomic DNA of *A. ustus* 3.3904 inserted in p3YG.
pLZ135 (p5HY-*opaC*)	a 1303 bp US PCR fragment of *opaC* from genomic DNA of *A. ustus* 3.3904 inserted in p5HY.
pLZ136 (p3YG-*opaC*)	a 1247 bp DS PCR fragment of *opaC* from genomic DNA of *A. ustus* 3.3904 inserted in p3YG.
pLZ137 (p5HY-*opaE*)	a 1391 bp US PCR fragment of *opaE* from genomic DNA of *A. ustus* 3.3904 inserted in p5HY.
pLZ138 (p3YG-*opaE*)	a 1231 bp DS PCR fragment of *opaE* from genomic DNA of *A. ustus* 3.3904 inserted in p3YG.
pLZ139 (p5HY-*opaF*)	a 1277 bp US PCR fragment of *opaF* from genomic DNA of *A. ustus* 3.3904 inserted in p5HY.
pLZ140 (p3YG-*opaF*)	a 1253 bp DS PCR fragment of *opaF* from genomic DNA of *A. ustus* 3.3904 inserted in p3YG.
pLZ61	*pYH-gpdA-opaB-pyrG*; a 2957 bp fragment of *opaB* with its terminator from genomic DNA of *A. ustus* 3.3904 inserted in *pYH-gpdA-pyrG*
pLZ62	pET-28a(+)-*opaC;* a 1595 bp fragment of *opaC* from cDNA of *A. ustus* 3.3904 with BamHI and EcoRI inserted in pET28a(+)
pLZ63	pET-28a(+)-*opaE;* a 825 bp fragment of *opaE* from cDNA of *A. ustus* 3.3904 with BamHI and EcoRI inserted in pET28a(+)

US: upstream; DS: downstream

Supplementary Table 4. Primers used in this study

Primers	**Sequence 5'-3'**	Targeted amplification
P5HY	CAAGACCAATGCGGAGCATATAC	2/3 of the *hph* resistance gene at the 5´-end from pUChph to construct p5HY
P3YG	GAATTGATTCCGGAAGTGCTTGAC	2/3 of the *hph* resistance gene at the 3´-end from pUChph to construct p3YG
p5HY-R	GCTGAAGTCGATTTGAGTCCAC	US of *hph* to verify 5F of *A. ustus* 3.3904 mutant
p3YG-F	GCATTAATGCATTGGACCTCGC	DS of *hph* to verify 3F of *A. ustus* 3.3904 mutant
opaA-U-F	ACCCTCACTAAAGGGCGGCCGCACTAGGTCTTTGTAGCGGGAGTTGTC	1453bp US fragment of *opaA* to construct pLZ131
opaA-U-R	AAGAATTGTTAATTAAGAGCTCAGATCCACTTCATGCATCGCACCTACA	
opaA-D-F	ACTCACTATAGGGCCCGGGCGTCGAAGCTCCCACAAGGATGACTGT	1427 bp DS fragment of *opaA* to construct pLZ132
opaA-D-R	TAGCCGCGGTACCAAGCTTACTCGAACATCTCCTAGCGTACATACGG	
opaA-F	ATCCAGAGGCCATTCAGATGG	1556 bp partial fragment of *opaA*
opaA-R	CCCGTGGATCCTGACGTAAAT	
opaA-5F-F	CGGGCATGATTCGCTTCACA	US of *hph* to verify △*opaA* mutant
opaA-3F-R	AGCCATGGATGTTGGCAGTC	DS of *hph* to verify △*opaA* mutant
opaB-U-F	AAGAATTGTTAATTAAGAGCTCAGATCGGTACCTTACAAATGTGGGGGT	1252 bp US fragment of *opaB* to construct pLZ133
opaB-U-R	ACCCTCACTAAAGGGCGGCCGCACTAGAGTGTCTGGTACTCCTGTTCG	
opaB-D-F	ACTCACTATAGGGCCCGGGCGTCGACACGGGTCAAGGTTGTTCAAT	1285 bp DS fragment of *opaB* to construct pLZ134
opaB-D-R	TAGCCGCGGTACCAAGCTTACTCGAATCTCCCGTTGTATCGGAGAG	
opaB-F	CCCGCGAGATCATGGATATCAT	1542 bp partial fragment of *opaB*
opaB-R	GCACGTATCCTGGTGAACATG	
opaB-5F-F	CCTCAGTCTCGAATGGCAATG	US of *hph* to verify △*opaB* mutant

Supplementary Table 4. (continued)

opaB-3F-R	TCGCTTCATCCAACTCCTGGT	DS of *hph* to verify △*opaB* mutant
opaC-U-F	AAGAATTGTTAATTAAGAGCTCAGATCAAGGATGGAAGCTGCGAGAT	1303 bp US fragment of *opaC* to construct pLZ135
opaC-U-R	ACCCTCACTAAAGGGCGGCCGCACTAGGCTGTGTCAGGCATTGGAATT	
opaC-D-F	ACTCACTATAGGGCCCGGGCGTCGATTTGGTGAGAATGGGGACAGG	1247 bp DS fragment of *opaC* to construct pLZ136
opaC-D-R	TAGCCGCGGTACCAAGCTTACTCGACTGGAATCACTCAAGGGGGAT	
opaC-F	TTTTCCCGTGCTTACTGAGGC	1486 bp partial fragment of *opaC*
opaC-R	AGCTTGAGAACCTCGAACCAT	
opaC-5F-F	TCTATTCGCGATCCATTCTGCG	US of *hph* to verify △*opaC* mutant
opaC-3F-R	CCCGCACAAGAGGAAATAACC	DS of *hph* to verify △*opaC* mutant
opaE-U-F	AAGAATTGTTAATTAAGAGCTCAGATCGTTACTCTATCCTAGCAACGCC	1391 bp US fragment of *opaE* to construct pLZ137
opaE-U-R	ACCCTCACTAAAGGGCGGCCGCACTAGCTTTTGCAAAGGTTGTCGGC	
opaE-D-F	ACTCACTATAGGGCCCGGGCGTCGAGGAGTGGTATGGTTACGACTTC	1231 bp DS fragment of *opaE* to construct pLZ138
opaE-D-R	TAGCCGCGGTACCAAGCTTACTCGACGAGCATGTAGGCACTGACATA	
opaE-F	CCGCAAGCTTGTCGACGGAGCTCGAATTCCTAGATTTCAATCCTTCCAC	825 bp partial fragment of *opaE*
opaE-R	TGGTGGACAGCAAATGGGTCGCGGATCCATGGGTCCTCTCCGTGTTTTC	
opaE-5F-F	GTGTGGTGCTGCCTTTGCTA	US of *hph* to verify △*opaE* mutant
opaE-3F-R	TGTTCCAGAAATTGCTCGTCCG	DS of *hph* to verify △*opaE* mutant
opaF-U-F	AAGAATTGTTAATTAAGAGCTCAGATCGACACGTGCTCTTACAGTGC	1277 bp US fragment of *opaF* to construct pLZ139
opaF-U-R	ACCCTCACTAAAGGGCGGCCGCACTAGCTATAAAGGTCGGAAGGCGAG	
opaF-D-F	ACTCACTATAGGGCCCGGGCGTCGAGAAGACTCGTACAGTGTGCCT	1253 bp DS fragment of *opaF* to construct pLZ140
opaF-D-R	TAGCCGCGGTACCAAGCTTACTCGAAAGGAAGTATCCCAGCTGCAG	

Supplementary Table 4. (continued)

opaF-F	TCTCTACGCGGAATCCAGCT	1448 bp partial fragment of *opaF*
opaF-R	CTCAGACGCGAAAGGTCATGA	
opaF-5F-F	CCCCATGGGTTGACCAATTAGG	US of *hph* to verify △*opaF* mutant
opaF-3F-R	AGTCTCCCCCATGGCTTAAAG	DS of *hph* to verify △*opaF* mutant
HE-opaB -F	CATCTTCCCATCCAAGAACCTTTAATCATGGCGGTTGCCCCCAATGTCT	2957 bp partial fragment of *opaB* from *A. ustus* 3.3904 to construct pLZ61
HE-opaB- R	TCGTCAGACACAGAATAACTCTCGCTAGTTGCGCATTAGGCGTCTGGCT	
pET-opaC- F	GCAAGCTTGTCGACGGAGCTCGAATTCCTAATTTTTTTCTTTCTCGTCG	1375 bp fragment of *opaC* *A. ustus* 3.3904 cDNA with was fused into pET28a(+) to construct pLZ62
pET-opaC- R	GGTGGACAGCAAATGGGTCGCGGATCCATGACTGTCCCCCAATACATCG	
pET-opaE- F	CCGCAAGCTTGTCGACGGAGCTCGAATTCCTAGATTTCAATCCTTCCAC	825 bp fragment of opaE from *A. ustus* 3.3904 cDNA was fused into pET28a(+) to construct pLZ63
pET-opaE- R	TGGTGGACAGCAAATGGGTCGCGGATCCATGGGTCCTCTCCGTGTTTTC	

US: upstream; DS: downstream

Supplementary Table 5. The ^{1}H and ^{13}C NMR spectroscopic data of oxepinamide F (1), E (2), and protuboxepin A (4)

	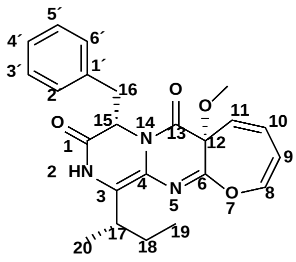 **1**		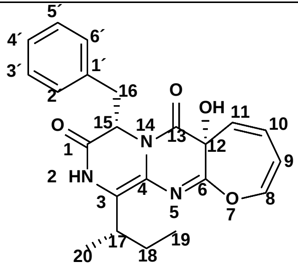 **2**		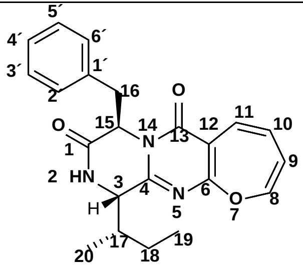 **4**	
solvents	DMSO-d_6		DMSO-d_6		$CDCl_3$	
Position	δ_H(multi., *J*)	δ_C	δ_H(multi., *J*)	δ_C	δ_H(multi., *J*)	δ_C
1		165.3		165.0		167.9
2	9.89, s		9.73, s		7.08, s	
3		123.4		122.1	2.64, d, 2.8	58.6
4		115.1		115.7		156.3
6		151.8		153.3		163.1
8	6.74, d, 7.3	144.4	6.72, d, 7.3	144.2	6.04, d, 5.8	143.3
9	5.48, t, 7.1	104.0	5.49, t, 7.1	104.1	5.64, t, 5.8	117.3
10	6.24, dd, 10.0, 7.0	130.8	6.18, dd, 10.2, 7.0	129.6	6.17, dd, 11.2, 5.8	128.1
11	5.77, d, 10.0	127.0	5.89, d, 10.2	130.0	6.78, d, 11.2	125.6
12		76.3		70.0		110.3
12-OCH_3 / 12-OH	2.77, s	52.3	6.34, s			
13		163.4		166.2		161.0
15	5.18, dd, 7.9, 5.7	55.8	5.03, dd, 6.9, 5.5	56.3	5.45, t, 4.7	57.0
16	2.88, m	36.4	2.96, dd, 13.7, 5.5	36.3	3.39 – 3.40, m	36.7
	3.12, dd, 13.7, 5.6		3.05, dd, 13.7, 6.9		3.39 – 3.40, m	
17	2.91, m	32.1	2.82, m	31.9	2.27, m	37.1
18	1.31, m	25.6	1.18, m	25.5	0.99 – 1.04, m	23.2
19	0.73, t, 7.4	12.1	0.62, t, 7.6	11.9	0.74, t, 7.5	12.0
20	0.95, d, 7.2	17.8	0.90, d, 7.3	17.5	0.85, d, 7.3	15.8
1´		135.1		135.4		134.4
2´, 6´	7.08, d, 6.9	129.6	7.06, dd, 8.2	129.3	6.94, dd, 8.4, 1.5	129.8
3´, 5´	7.24, t, 7.3	128.3	7.24, tt, 7.0, 1.6	128.2	7.24, tt, 7.0, 1.5	129.0
4´	7.18, d, 7.3	126.8	7.20,d,7.1	126.7	7.30, t, 7.0	128.2

The NMR data of **1**, **2**, and **4** correspond well to those of oxepinamide F,[1] oxepinamide E,[1] and protuboxepin A,[2] respectively

Supplementary Table 6. The ^{1}H and ^{13}C NMR spectroscopic data with key HMBC, COSY, and NOESY correlations of protuboxepin K (3)

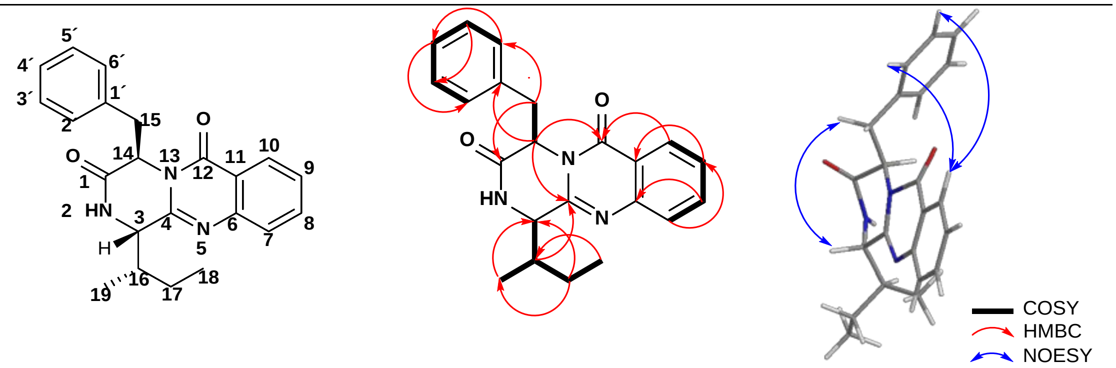

solvent	CDCl$_3$			
Position	δ_H(multi., *J*)	δ_C	HMBC	NOESY
1		168.8		
3	2.76, d, 2.2	58.4	C-4, C-16, C-17, C-19	H-15, H-16, H-19
4		150.3		
6		147.1		
7	7.62, br d, 8.3	127.5	C-6, C-8, C-9, C-11	H-8, H-9, H-10
8	7.78, ddd, 8.3, 7.2, 1.2	134.8	C-6, C-10, C-11	H-7, H-9, H-10
9	7.52, ddd, 8.0, 7.2, 1.2	127.2	C-6, C-7, C-8, C-11	H-7, H-8, H-10
10	8.34, dd, 8.0, 1.2	126.9	C-6, C-7, C-8, C-12	H-8, H-9, H-2´,6´, H-3´,5´
11		120.1		
12		160.9		
14	5.64, br t, 4.7	57.1	C-1, C-4, C-1´, C-15, C-12	H-15, H-2´,6´
15	3.47, dd, 13.8, 5.5	37.3	C-1, C-1´, C-14, C-2´,6´	H-3, H-14, H-2´,6´
	3.44, dd, 13.8, 4.0			
16	2.47, m	36.5	C-4, C-17, C-18, C-19	H-3, H-17, H-18, H-19
17	1.19, m	23.3	C-3, C-16, C-18, C-19	H-16, H-18, H-19
	1.04, m			H-16, H-18, H-19
18	0.77, t, 7.4	12.3	C-16, C-17	H-16, H-17, H-19
19	0.91, d, 7.3	15.8	C-3, C-16, C-17	H-3, H-16, H-17, H-18
1´		135.1		
2´, 6´	6.92, dd, 8.2, 1.4	129.9	C-1, C-4, C-1´, C-4´, C-2´,6´, C-14, C-15	H-10, H-14, H-15, H-3´,4´,5´
3´, 5´	7.18, t, 7.5	128.8	C-1´, C-3´,5´	H-10, H-2´,4´,6´
4´	7.27, t, 7.5	128.0	C-1´, C-2´,6´	H-2´,6´, H-3´,5´

The NMR data of **3** correspond well to those of protuboxepin K, published recently by Ohte *et al*.[3]

Supplementary Table 7. The ^{1}H and ^{13}C NMR spectroscopic data with key HMBC, COSY, and NOESY correlations of 15-*epi*-oxepinamide E (5)

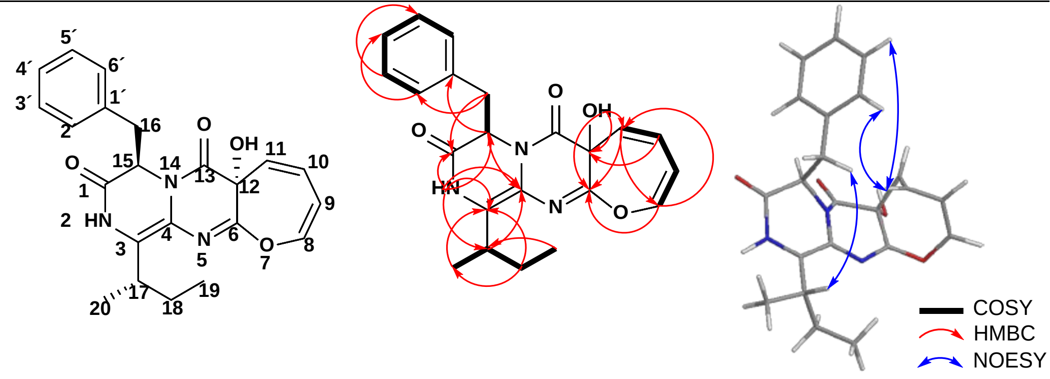

solvent	DMSO-d$_6$			
Position	δ_H(multi., *J*)	δ_C	HMBC	NOESY
1		164.9		
2	9.64, s		C-1, C-3, C-4, C-15, C-17	H-20, H-3´,4´,5´
3		120.7		
4		116.2		
6		153.5		
8	6.64, d, 7.2	144.0	C-6, C-10, C-9, C-11	H-9, H-10, H-11, H-2´,6´, H-3´,5´
9	5.49, t, 7.2	105.3	C-8, C-11	H-8, H-10, H-11, H-2´,6´, H-3´,5´
10	6.12, dd, 10.4, 6.8	128.6	C-8, C-12	H-8, H-9, H-2´,6´, H-3´,5´
11	5.70, d, 10.4	131.5	C-6, C-8, C-9, C-12, C-13	H-8, H-9, H-15
12-(OH)	6.52, s	69.6	C-6, C-11, C-12	
13		165.5		
15	5.19, t, 5.1	55.7	C-1, C-1´, C-4, C-16	H-11
16	3.01, dd, 13.7, 5.3	36.5	C-1, C-1´, C-2´,6´, C-15	H-17
	3.13, dd, 13.7, 5.0			H-17
17	2.82, m	31.5	C-3, C-4, C-18, C-19, C-20	H-16, H-18, H-19, H-20
18	1.40, m	25.7	C-3, C-17, C-19, C-20	H-17, H-18b, H-19, H-20
	1.30, m			H-17, H-18a, H-19, H-20
19	0.67, t, 7.4	11.9	C-17, C-18	H-17, H-18, H-20
20	0.63, d, 7.1	17.0	C-3, C-17, C-18	HN-2, H-17, H-18, H-19
1´		135.0		
2´, 6´	7.00, dd, 7.0, 2.9	129.6	C-4´, C-2´,6´, C-16	H-8, H-9, H-10, H-3´,4´,5´
3´, 5´	7.20, m	128.1	C-1´, C-3´,5´, C-2´,6´	HN-2, H-8, H-9, H-10, H-2´,6´
4´	7.20, m	126.8	C-1´, C-2´,6´	HN-2, H-10, H-2´,6´H-3´,5´

Supplementary Table 8. The ^{1}H and ^{13}C NMR spectroscopic data with key HMBC, COSY, and NOESY correlations of 15-*epi*-oxepinamide F (6)

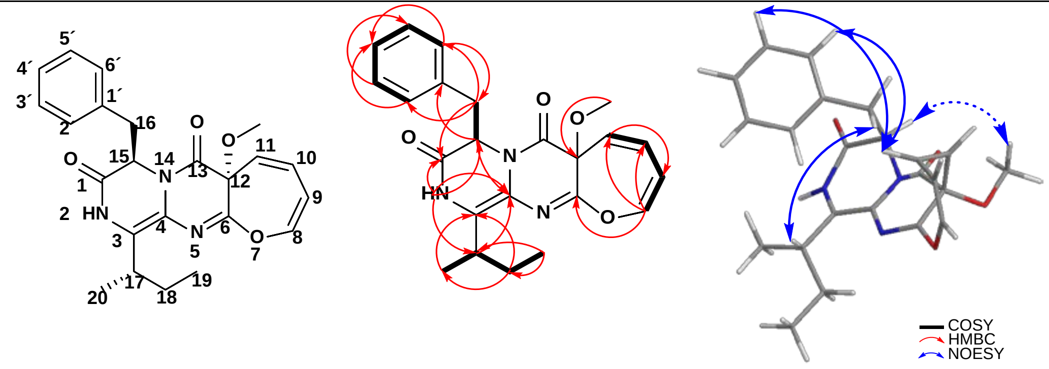

solvent	DMSO-d$_6$			
Position	δ_H(multi., *J*)	δ_C	HMBC	NOESY
1		165.0		
2	9.75, s		C-1, C-3, C-4, C-15, C-17	H-18, H-20, H-3´,5´, H-2´,6´, H-4´
3		122.0		
4		115.9		
6		151.3		
8	6.67, d, 7.2	144.3	C-6, C-10, C-9, C-11	H-9, H-10, H-11, H-2´, 6´
9	5.50, t, 7.2	105.4	C-8, C-10, C-11	H-8, H-10, H-11, OCH$_3$-12, H-15
10	6.21, dd, 10.4, 6.8	128.7	C-8, C-12	H-8, H-9, H-11, H-2´,6´, H-3´,5´
11	5.64, d, 10.4	130.0	C-6, C-8, C-9, C-12, C-13	H-8, H-9, H-10, H-15
12		76.1		
12-OCH$_3$	3.11, s	52.8	C-12	H-9, H-15
13		163.0		
15	5.24, t, 5.2	55.9	C-1, C-1´, C-4, C-13, C-16	H-9, H-11, OCH$_3$-12, H-16a
16	3.01, dd , 14.1, 5.3	36.4	C-1, C-1´, C-2´,6´, C-15	H-15, H-16b, H-17
	3.14, dd, 14.1, 5.1			H-16a, H-17
17	2.84, m	31.6	C-3, C-18, C-20	H-16a, H-16b, ,H-18a, H-20
18	1.41, m	25.8	C-17, C-19	HN-2, H-17, H-18b, H-19, H-20
	1.30, m		C-3, C-17, C-19, C-20	HN-2, H-18a, H-19, H-20
19	0.66, t, 7.4	11.7	C-3, C-17, C-18	H-18, H-20
20	0.66, d, 7.0	16.9	C-3, C-17, C-18	HN-2, H-17, H-18, H-19
1´		134.9		
2´, 6´	6.99, dd, 7.0, 3.0	129.6	C-4´, C-2´,6´, C-16	HN-2, H-8, H-10, H-3´,5´, H-4´
3´, 5´	7.20, m	128.2	C-1´, C-3´,5´, C-2´,6´	HN-2, H-10, H-2´,6´, H-4´
4´	7.20, m	126.8	C-1´, C-2´,6´	HN-2, H-10, H-2´,6´, H-3´,5´

Supplementary Table 9. Conserved motifs in P450 enzymes used in Weblogo analysis for OpaB

Entries	Conserved sequence	Entries	Conserved sequence	Entries	Conserved sequence
1. ACM47223.1_8-506	AVIKETLRIS	33. KAF3893027.1_16-495	AVVKESLRLS	65. RYP76854.1_33-503	AVMKESLRLA
2. ADY18333.1_21-506	AVIKETLRIS	34. KAF4773391.1_24-504	AVIKEILRLG	66. SCV45835.1_49-420	AVIHETLRIS
3. AET10042.1_8-506	AVIKETLRIS	35. KIA75457.1_1-575	AVIRETMRLL	67. TEY57352.1_27-515	AAVKESMRIH
4. AEV21239.1_8-506	AVIKETLRIS	36. KIA75687.1_41-518	AIIKESLRLY	68. TGJ85573.1_7-412	ATIKEILRLA
5. AGA37280.1_48-520	AVLKETMRLS	37. KLP01168.1_49-420	AVIHETLRIS	69. TGO15980.1_44-505	AVVKESMRVH
6. AGN89898.1_8-506	AVIKETLRIS	38. KPA41271.1_49-423	AVINETLRIS	70. TGO83795.1_21-421	AVVKESMRVY
7. AGS31995.1_8-506	AVIKETLRIS	39. KXG49075.1_25-413	GVIRETMRLA	71. THC90386.1_21-501	AVVKETLRMY
8. ANY57880.1_1-438	AVVKETLRLY	40. KXH34948.1_24-504	AVIKEILRLG	72. THV46788.1_67-578	AVVKESMRVH
9. CEL05736.1_45-421	AVIKETHRMW	41. KXH37991.1_15-507	AVIKEILRLG	73. TRX96397.1_25-409	AAIKEMIRLA
10. CEO59279.1_38-411	AIIKEAHRIW	42. KXH39310.1_24-504	AVIKEILRLG	74. XP_001267636.1_26-411	AVIKEAHRMW
11. EGD97062.1_33-512	AVVKESLRLS	43. KZL82384.1_37-422	ACIKESLRMS	75. XP_015409727.1_24-402	AVIKETLRVS
12. EGE01905.1_33-512	AVVKESLRLS	44. OJI99626.1_8-543	AVIRESMRLY	76. XP_016598809.1_33-416	AVIKEALRLS
13. EMR81817.1_1-438	AVVKESMRIH	45. OJJ05850.1_14-509	AVVKETLRLS	77. XP_018060973.1_14-510	AVVKESLRLS
14. EQB44036.1_2-504	ATIKEILRLS	46. OJJ57312.1_40-512	AVVKETLRLS	78. XP_020056257.1_2-494	AVIKEALRMS
15. EXF79469.1_15-402	AVIKEILRLG	47. OOQ90409.1_38-411	AIIKEAHRIW	79. XP_022388567.1_14-504	AVIEEVLRIS
16. EZF29424.1_16-495	AVVKESLRLS	48. OOQ91747.1_19-514	ATVKESLRLS	80. XP_022470328.1_15-505	AVIKEILRLG
17. GAO81247.1_31-521	AVVKECLRLS	49. OQD98286.1_24-412	AVIRETMRVA	81. XP_023426771.1_49-420	AVIHETLRIS
18. GAO87325.1_29-521	AVIKETMRLY	50. OQE41206.1_33-522	AVIKEALRLS	82. XP_024548999.1_26-516	AVVKESMRIH
19. GAO88330.1_104-491	AVIKETHRMW	51. OQE41255.1_24-412	GVIRETMRLA	83. XP_024678521.1_46-521	AVVKESLRLS
20. GAW23203.1_12-418	ATIKEILRLA	52. OTA60878.1_17-500	AVVKEALRLS	84. XP_025429720.1_6-502	AVIKESLRMS
21. GBF60059.1_16-495	AVVKESLRLS	53. OTA61602.1_19-501	AVIKESLRLS	85. XP_025434452.1_47-509	AVIKETLRLS
22. GFF33539.1_29-518	AVIKETMRLY	54. OTA81174.1_25-500	AVVEETLRLS	86. XP_025447741.1_31-508	AIIKETMRIS
23. GFF48416.1_46-521	AVVKECLRLS	55. OTA98483.1_29-501	AVIKESLRLS	87. XP_025487304.1_10-453	AVIKETLRLS
24. GFF51631.1_31-523	AVVKECLRLS	56. OTB14165.1_14-489	AVIKETLRLS	88. XP_025502623.1_31-508	AIIKETMRIS
25. GFF92884.1_46-521	AVVKECLRLS	57. POS72677.1_32-504	ACIKESLRMS	89. XP_026601988.1_19-498	AVVKETLRLA
26. KAB8069486.1_31-399	AVIKEALRIS	58. RAK81184.1_20-508	AIIKETMRIS	90. XP_026602854.1_14-530	AVIKETLRLY
27. KAB8073414.1_5-387	AVIKEALRIS	59. RHZ53947.1_35-408	AVIKESLRLS	91. XP_026613670.1_71-448	AIIKEAHRIW
28. KAE8351816.1_19-501	AVVKETLRLS	60. RLL97463.1_15-497	AVIKETLRLI	92. XP_026617163.1_53-522	AVVKECLRLS
29. KAF0324645.1_2-504	ATIKEILRLS	61. RWA13246.1_12-418	ATIKEILRLA	93. XP_031881851.1_2-504	ATIKEILRLS
30. KAF2971667.1_65-440	ATIKEILRLA	62. RYC59098.1_7-408	AAVKEMIRLA	94. XP_033423998.1_21-503	AVVKETLRMY
31. KAF3761239.1_6-482	AVIKESLRVS	63. RYP24796.1_5-406	AVMKESLRLA	95. XP_664089.1_6-392	AIIKESHRIW
32. KAF3809983.1_2-513	ATIKEILRLS	64. RYP66740.1_19-400	AVIKEILRLS	96. XP_682494.1_48-464	AIIKETLRLY

Supplementary Figures

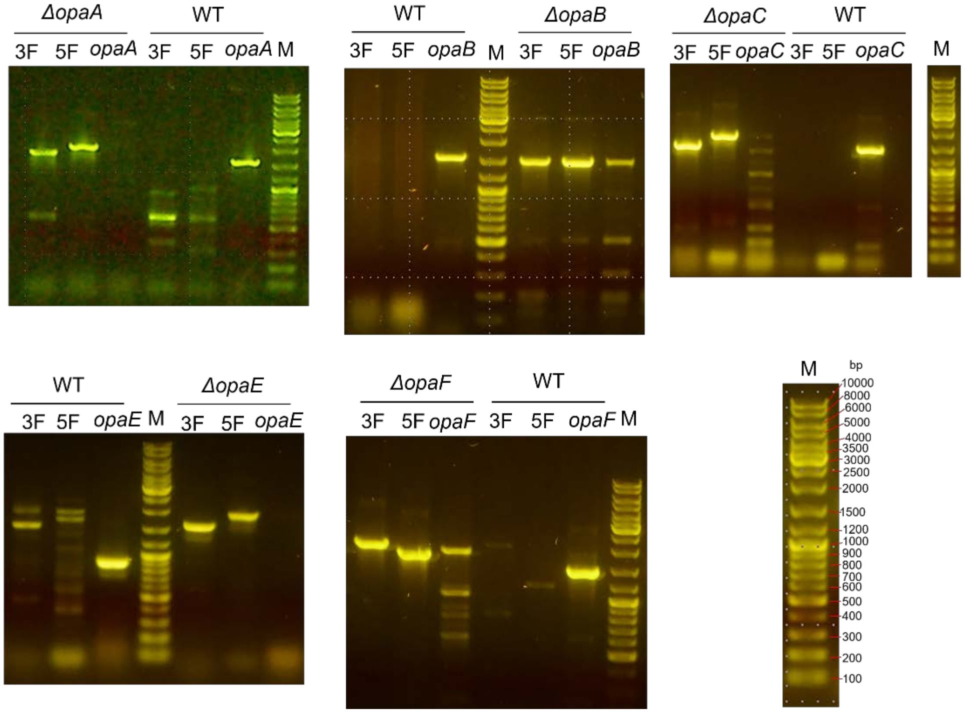

Supplementary Figure 1. PCR verification of deletion mutants of *A. ustus* 3.3904.
PCR amplification for three different fragments from genomic DNA of WT and deletion mutants was used to prove the presence/absence of the target gene and the integration site of the selection marker with up- and downstream regions. The PCR primers are given in Table S4. All the experiments were repeated twice.

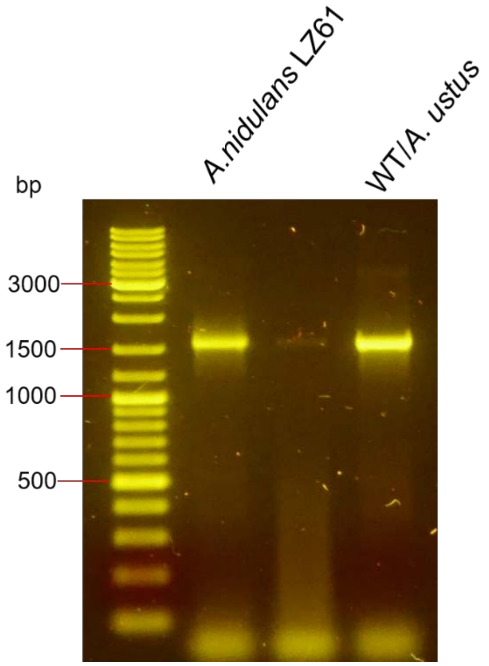

Supplementary Figure 2. PCR verification of *opaB* heterologous expression transformant
A. nidulans-pYH-*gpdA*-*opaB*-*pyrG* (HE-*opaB*). A fragment of 1.5 kb within the target gene was amplified from the primers listed in Table S4. The experiments were repeated twice.

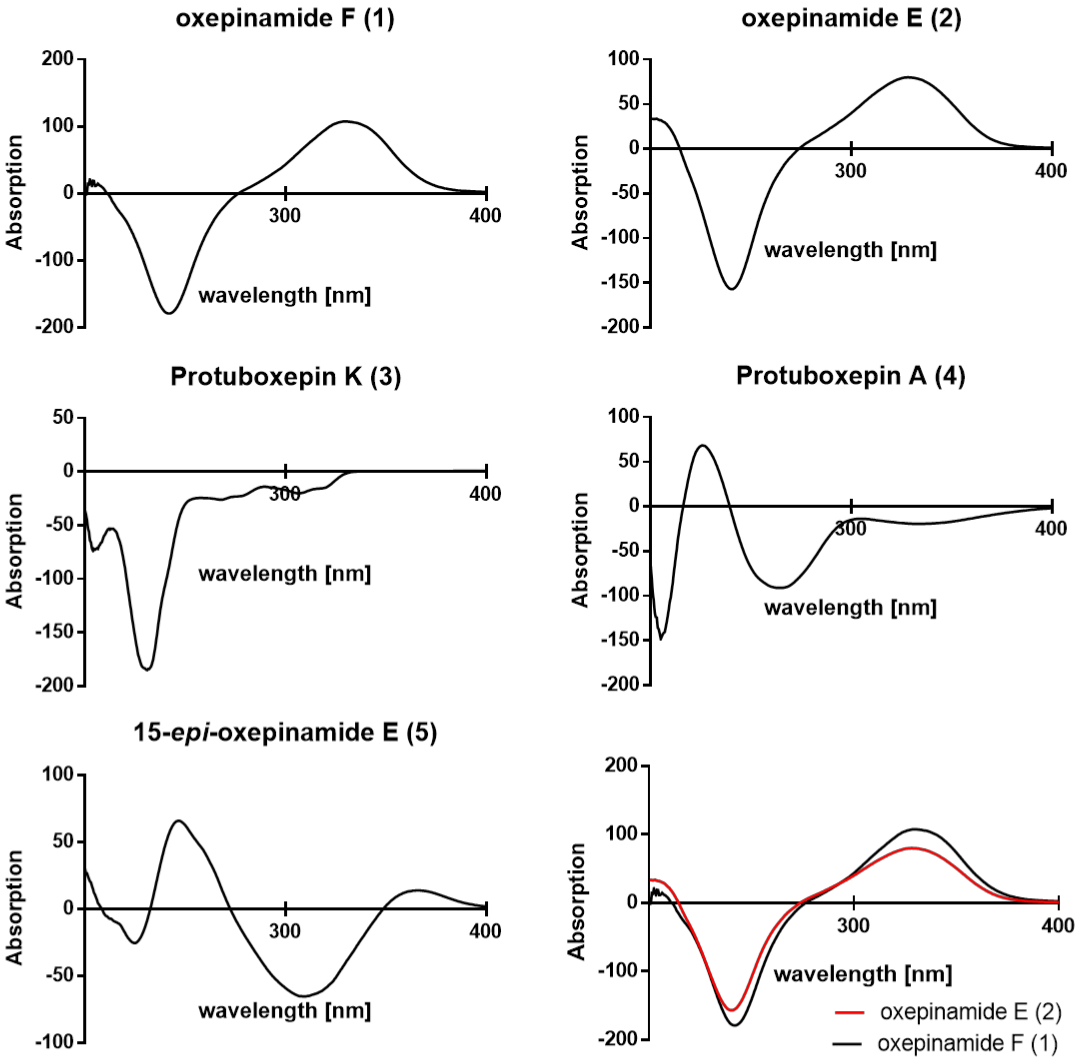

Supplementary Figure 3. CD spectra of compounds 1 – 5 in CH_3OH (200 – 400 nm)

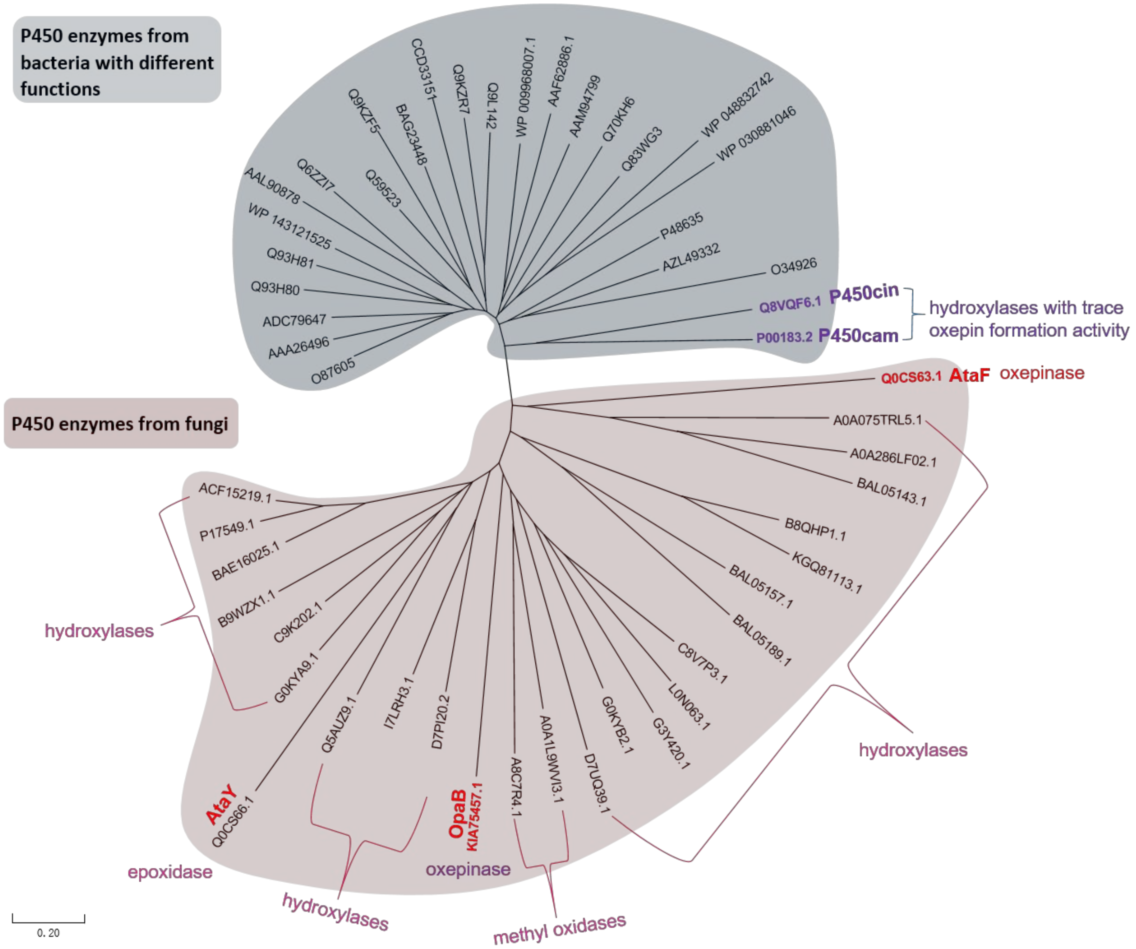

Supplementary Figure 4. Phylogenetic analysis of OpaB and functionally characterized P450s from bacteria and fungi. The protein sequences were downloaded from NCBI database.

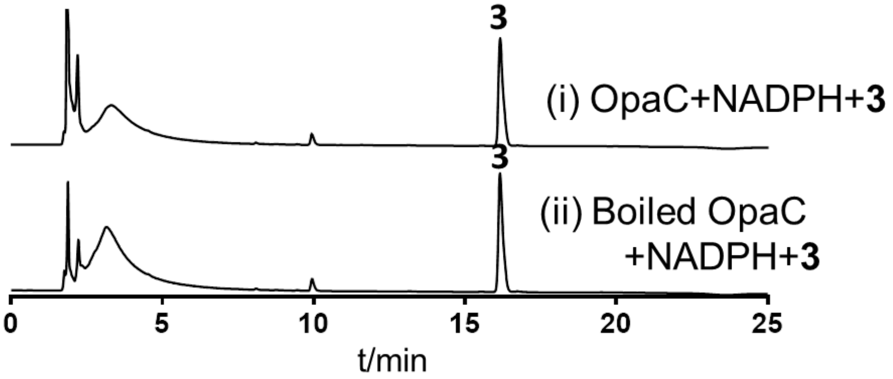

Supplementary Figure 5. HPLC analysis of the incubation mixtures of OpaC with protuboxepin K (**3**)

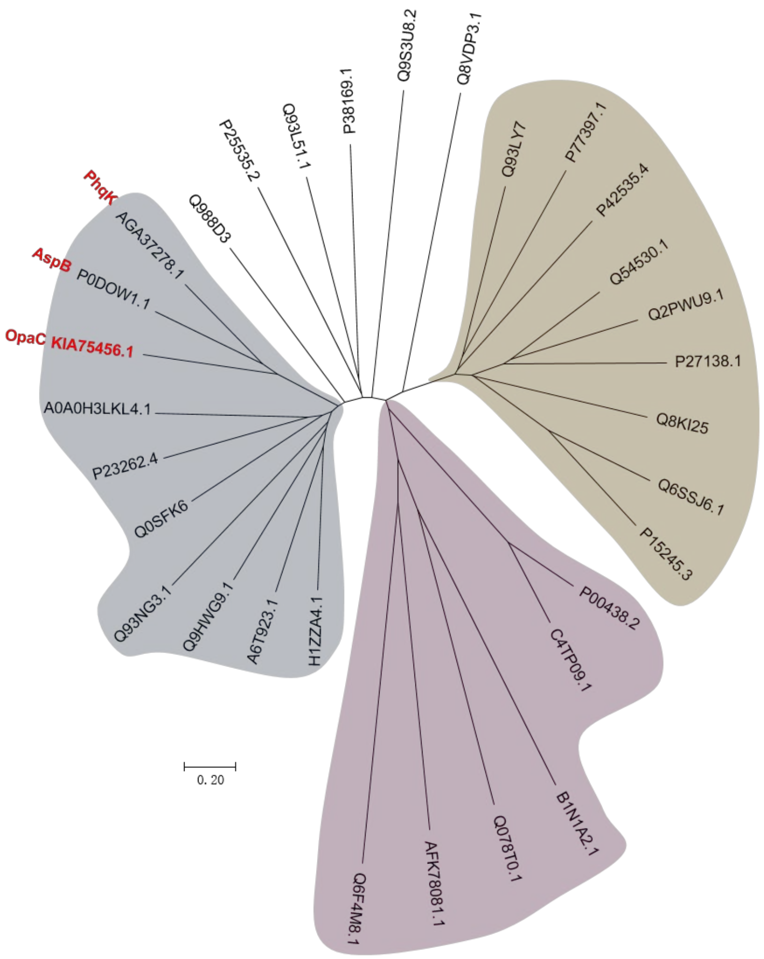

Supplementary Figure 6. Phylogenetic analysis of class A flavin monooxygenases. OpaC, AspB and PhqK are highlighted in red. The protein sequences were downloaded from NCBI database.

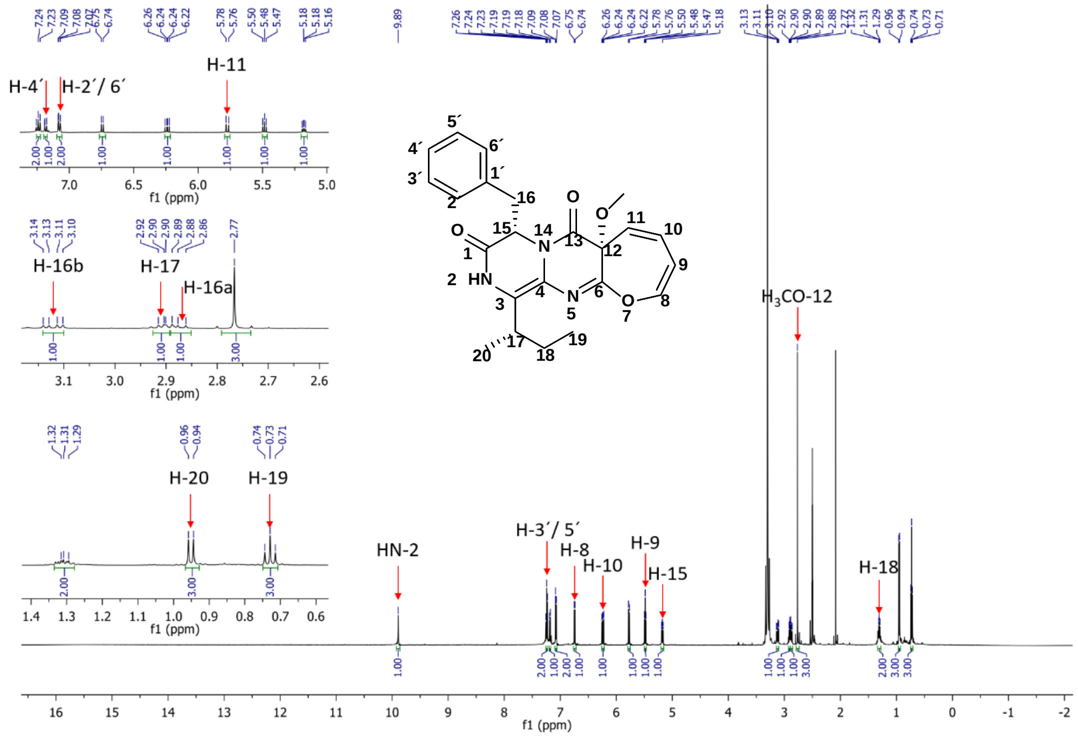

Supplementary Figure 7. **^{1}H NMR spectrum of oxepinamide F (1) in DMSO-d_6 (500 MHz)**

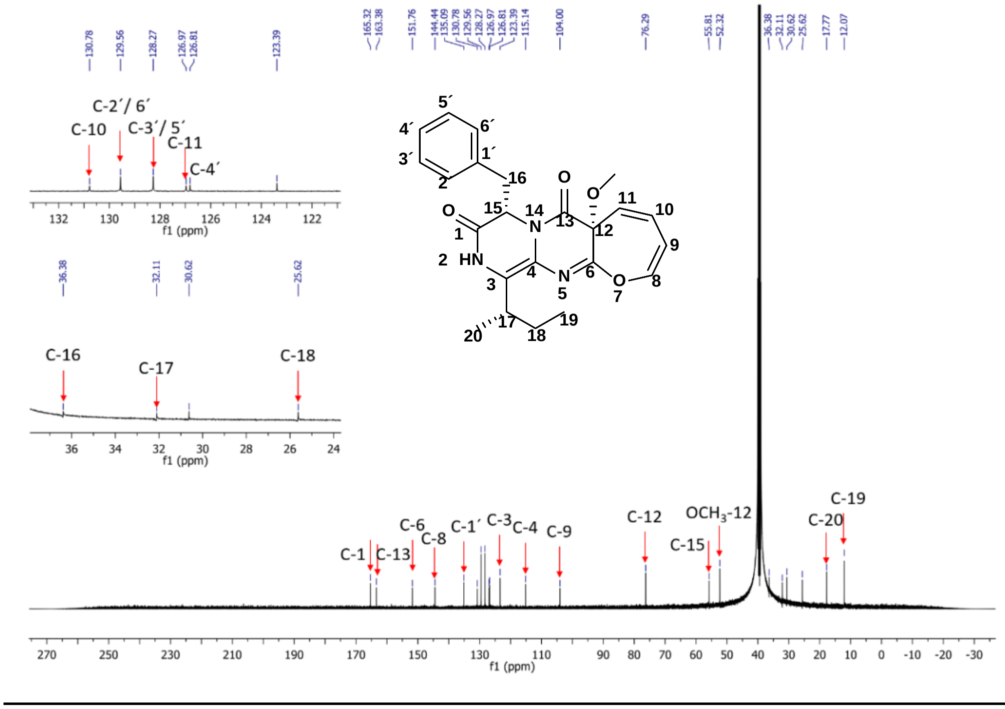

Supplementary Figure 8. **$^{13}C\{^{1}H\}$ NMR spectrum of oxepinamide F (1) in DMSO-d_6 (125 MHz)**

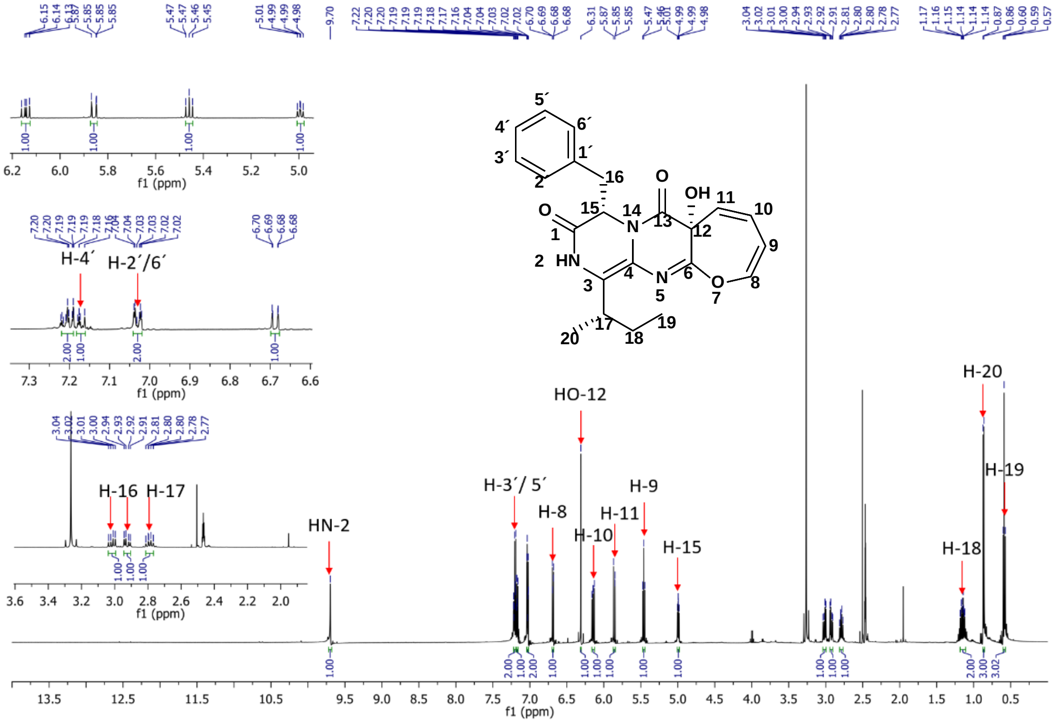

Supplementary Figure 9. ^{1}H NMR spectrum of oxepinamide E (2) in DMSO-d_6 (500 MHz)

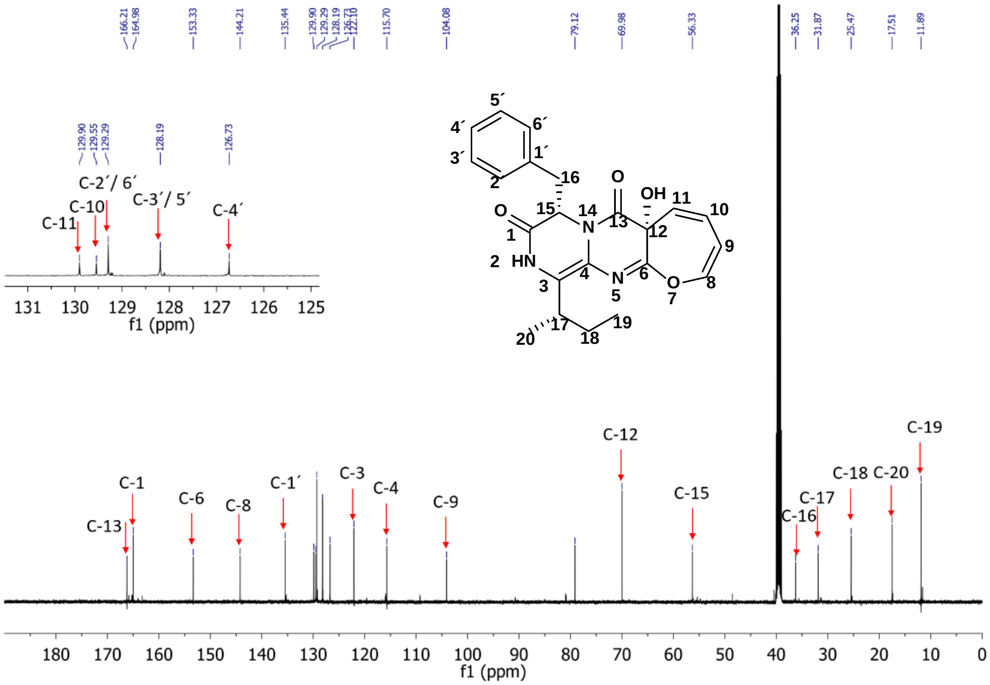

Supplementary Figure 10. $^{13}C\{^{1}H\}$ NMR spectrum of oxepinamide E (2) in DMSO-d_6 (125 MHz)

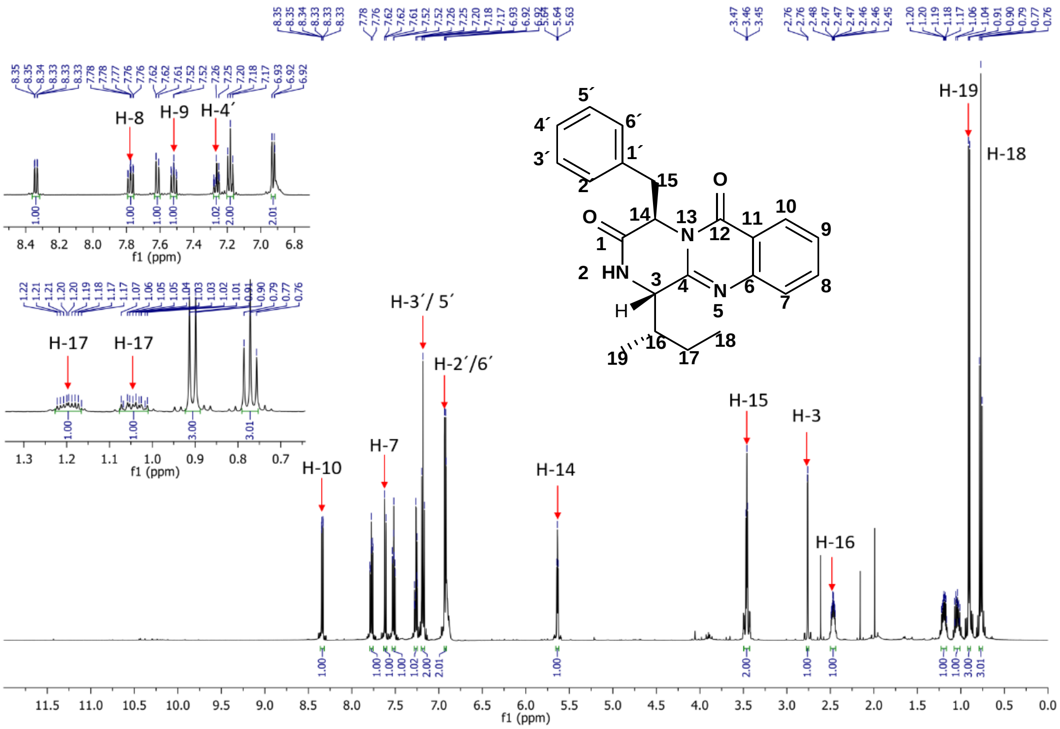

Supplementary Figure 11. **^{1}H NMR spectrum of protuboxepin K (3) in $CDCl_3$ (500 MHz)**

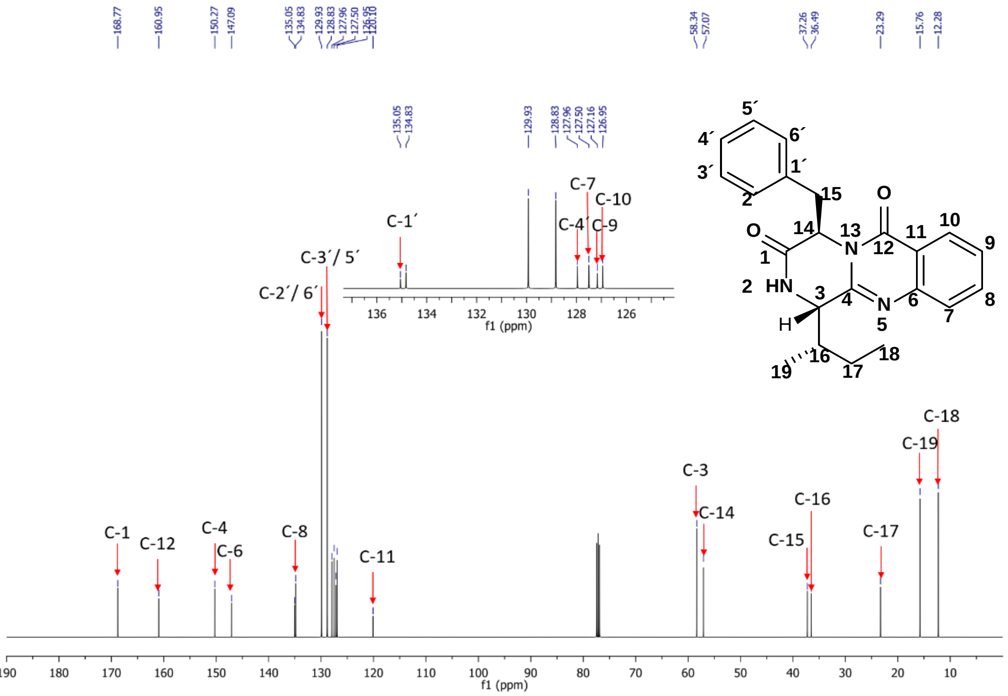

Supplementary Figure 12. **$^{13}C\{^{1}H\}$ NMR spectrum of protuboxepin K (3) in $CDCl_3$ (125 MHz)**

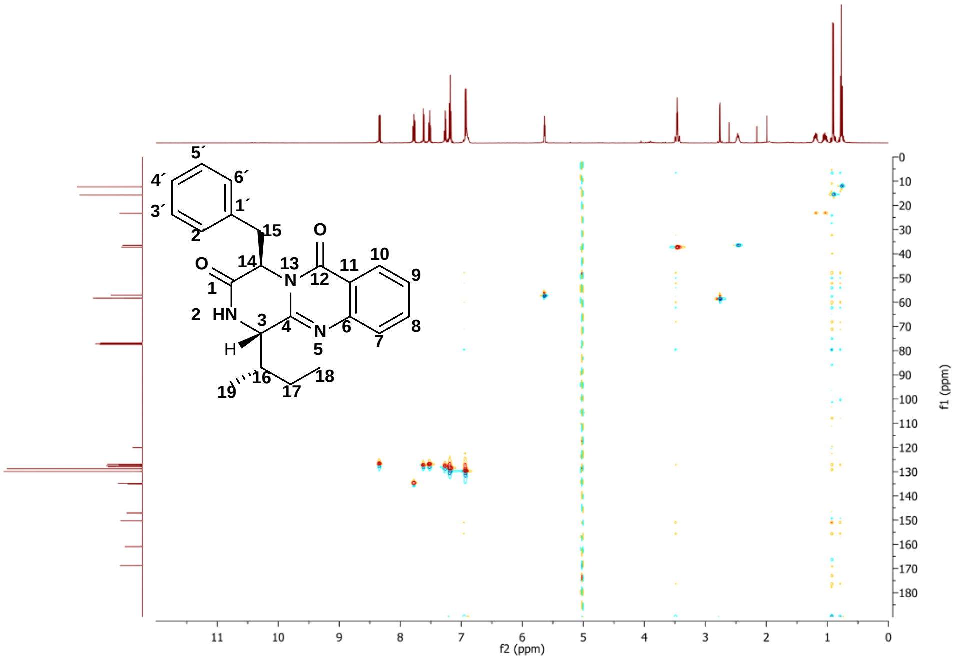

Supplementary Figure 13. HSQC spectrum of protuboxepin K (3) in $CDCl_3$

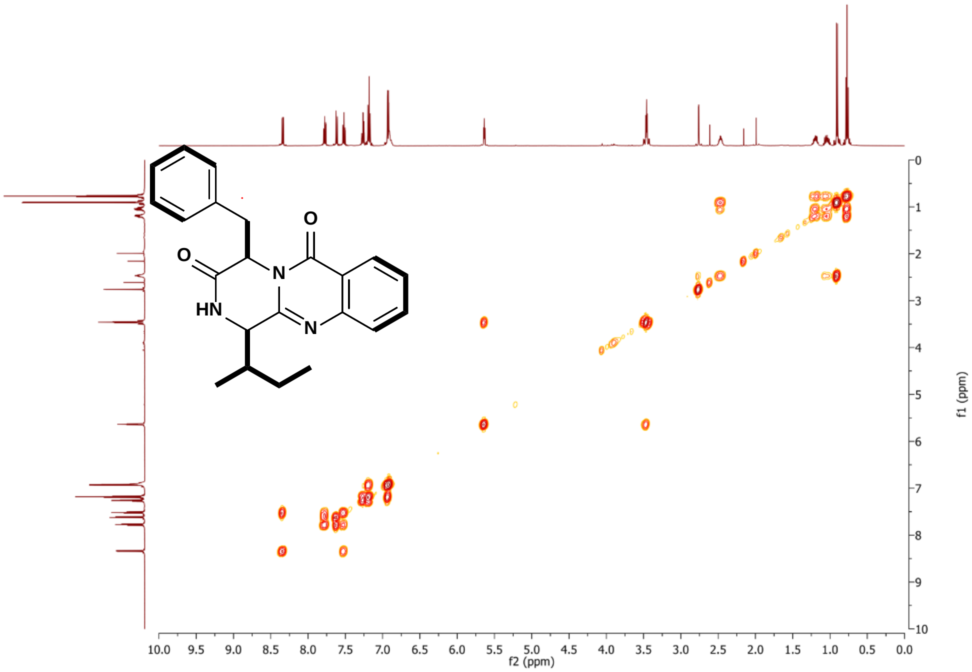

Supplementary Figure 14. COSY spectrum of protuboxepin K (3) in $CDCl_3$

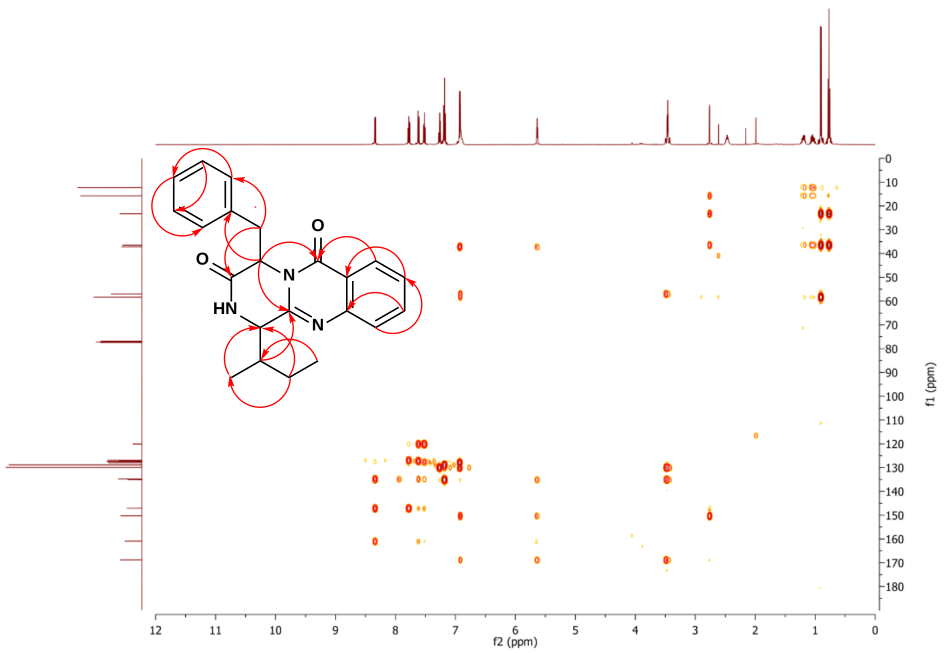

Supplementary Figure 15. HMBC spectrum of protuboxepin K (3) in $CDCl_3$

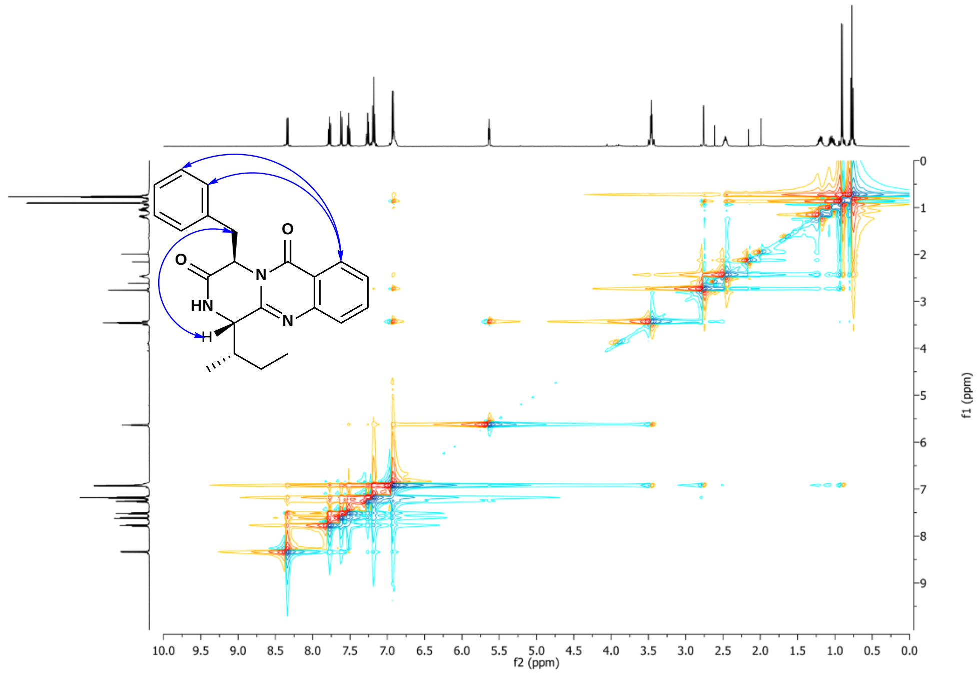

Supplementary Figure 16. NOESY spectrum of protuboxepin K (3) in $CDCl_3$

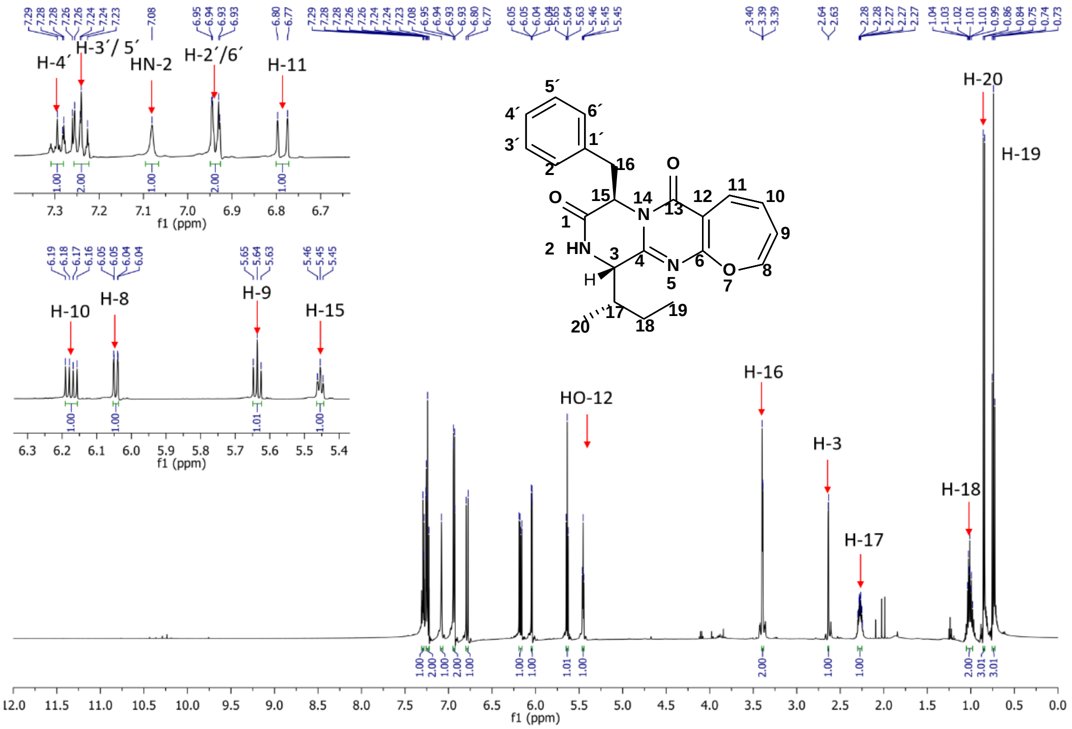

Supplementary Figure 17. ^{1}**H NMR spectrum of protuboxepin A (4) in** $CDCl_3$ **(500 MHz)**

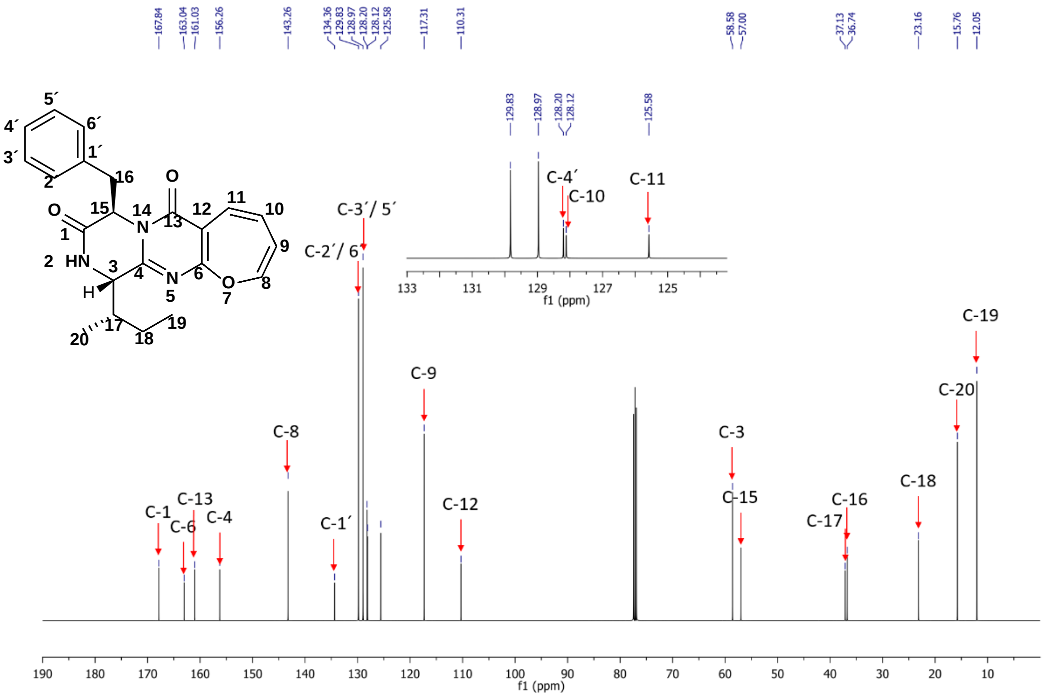

Supplementary Figure 18. $^{13}C\{^{1}H\}$ **NMR spectrum of protuboxepin A (4) in** $CDCl_3$ **(125 MHz)**

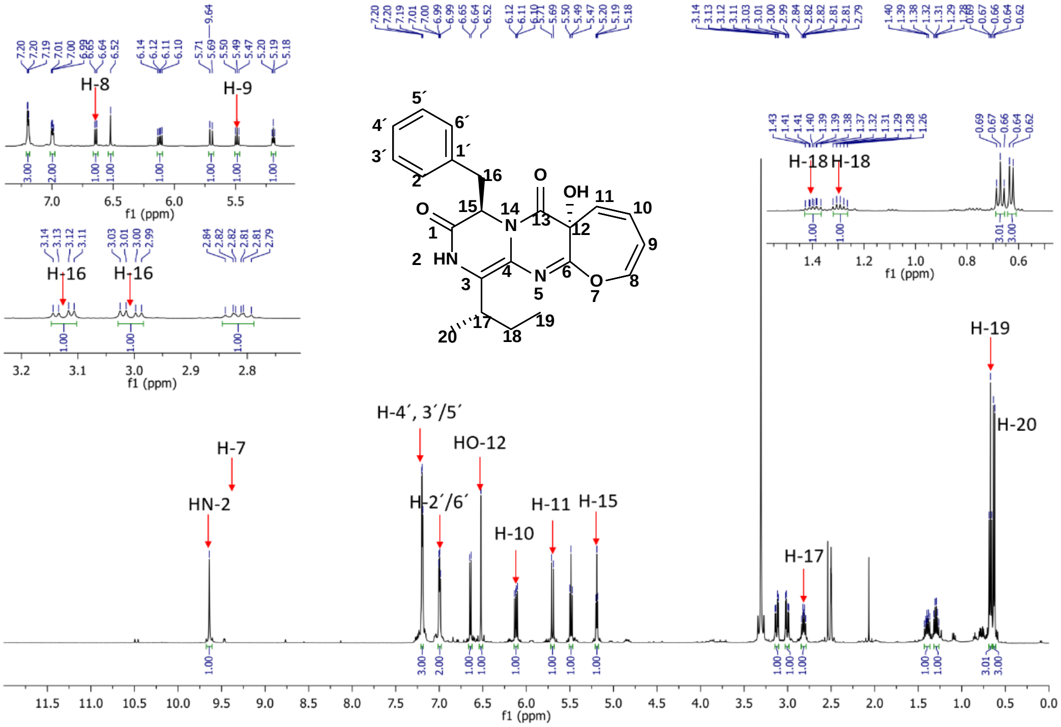

Supplementary Figure 19. ^{1}**H NMR spectrum of 15-*epi*-oxepinamide E (5) in DMSO-d$_6$ (500 MHz)**

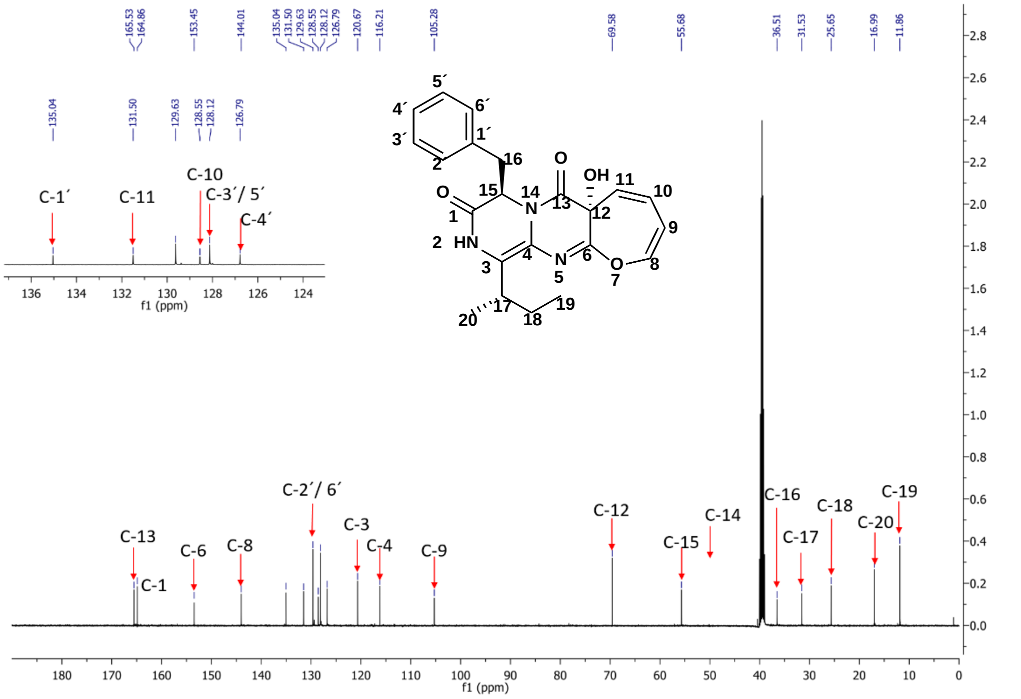

Supplementary Figure 20. ^{13}**C{**^{1}**H} NMR spectrum of 15-*epi*-oxepinamide E (5) in DMSO-d$_6$ (125 MHz)**

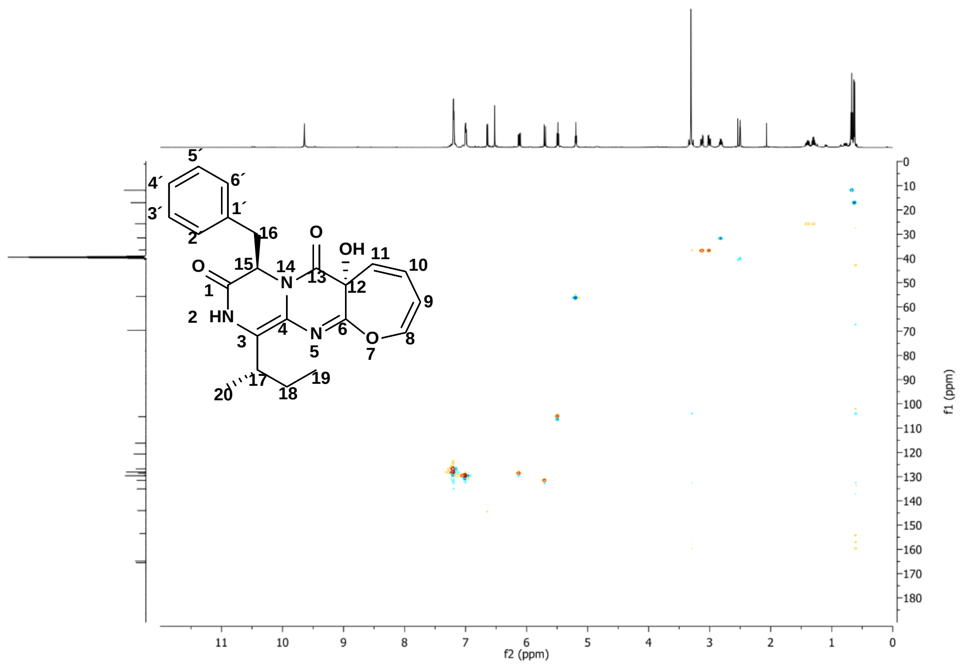

Supplementary Figure 21. HSQC spectrum of 15-*epi*-oxepinamide E (5) in DMSO-d$_6$

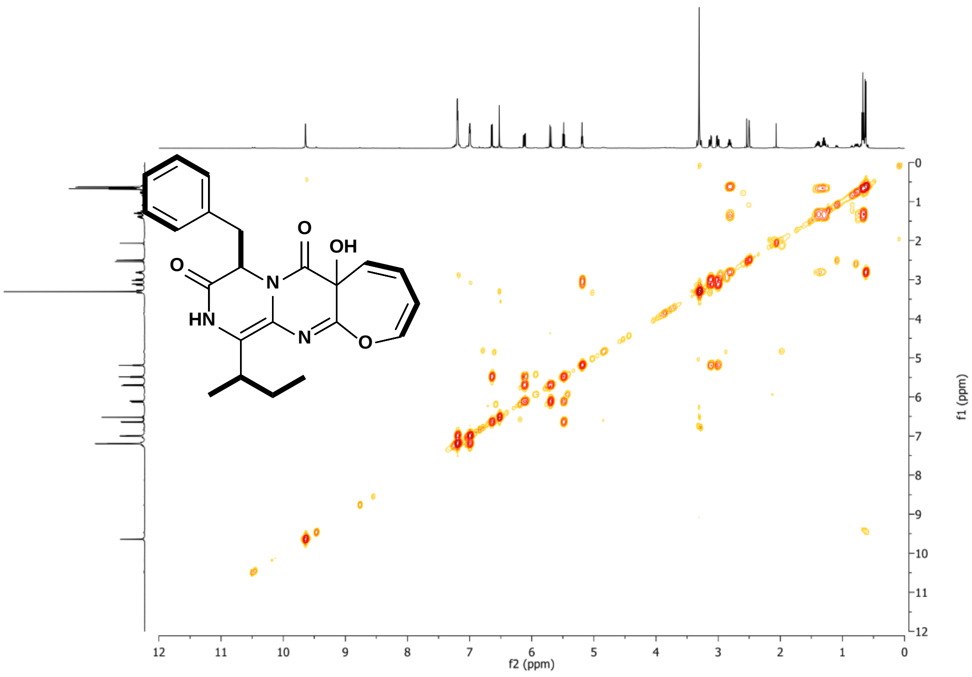

Supplementary Figure 22. COSY spectrum of 15-*epi*-oxepinamide E (5) in DMSO-d$_6$

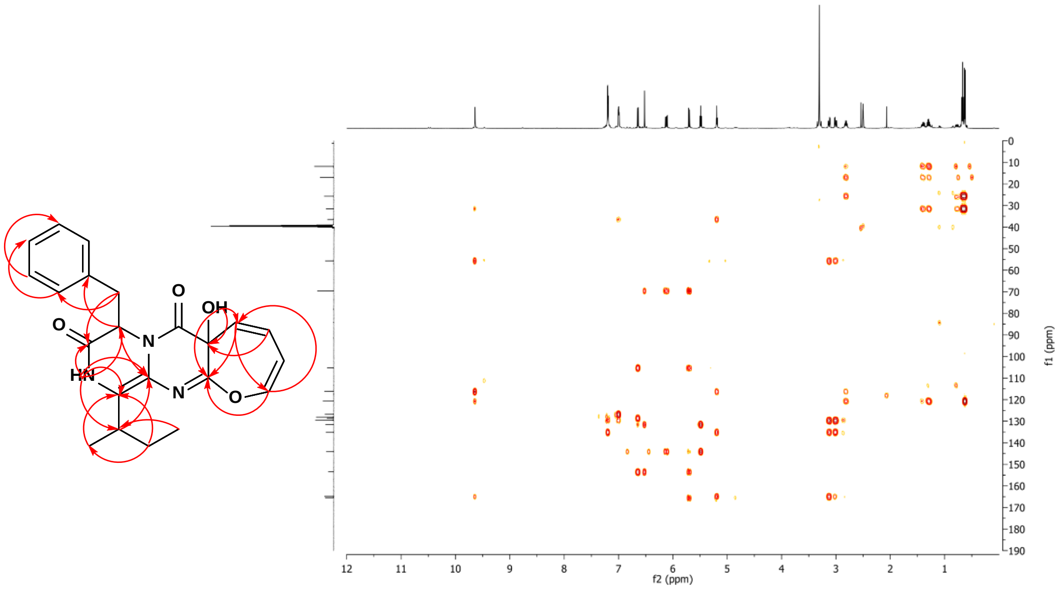

Supplementary Figure 23. HMBC spectrum of 15-*epi*-oxepinamide E (5) in DMSO-d$_6$

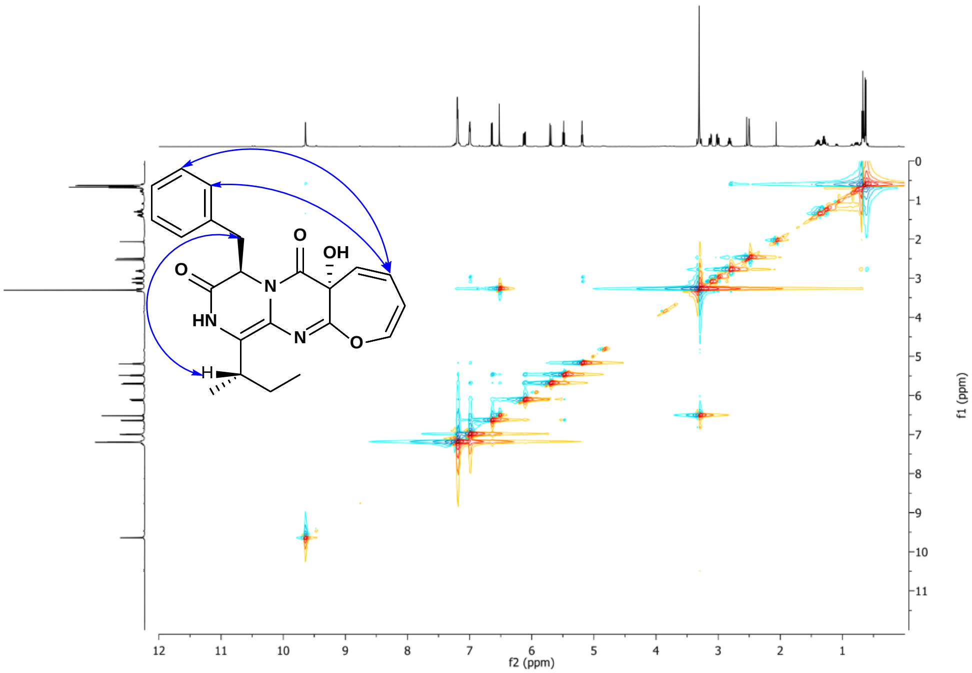

Supplementary Figure 24. NOESY spectrum of 15-*epi*-oxepinamide E (5) in DMSO-d$_6$

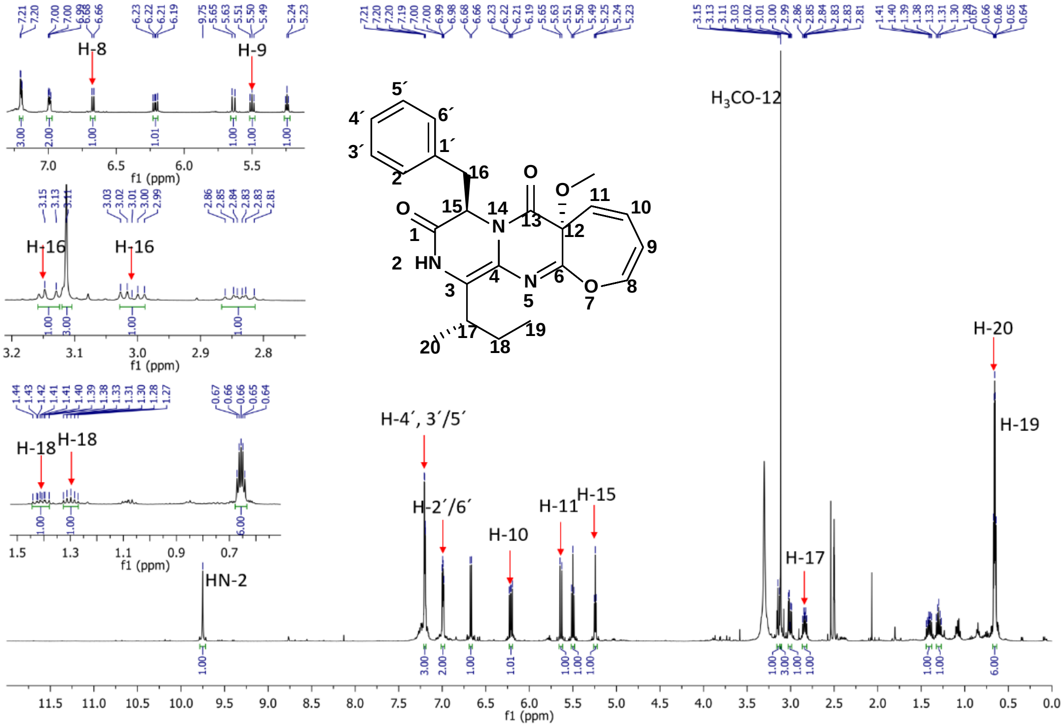

Supplementary Figure 25. **^{1}H NMR spectrum of 15-*epi*-oxepinamide F (6) in DMSO-d$_6$ (500 MHz)**

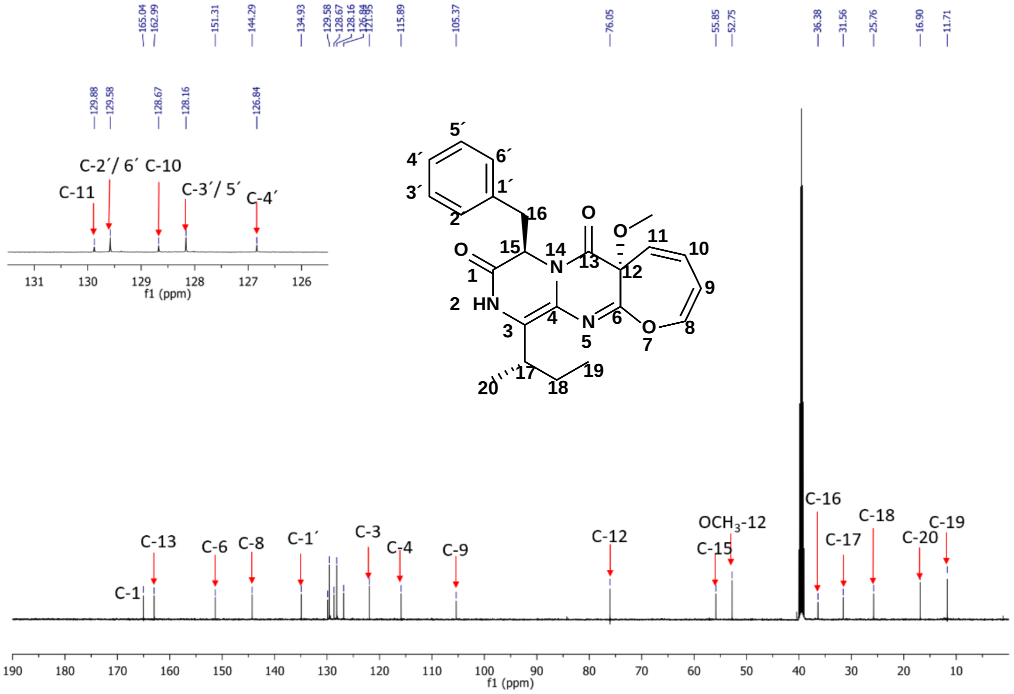

Supplementary Figure 26. **^{13}C{^{1}H} NMR spectrum of 15-*epi*-oxepinamide F (6) in DMSO-d$_6$ (125 MHz)**

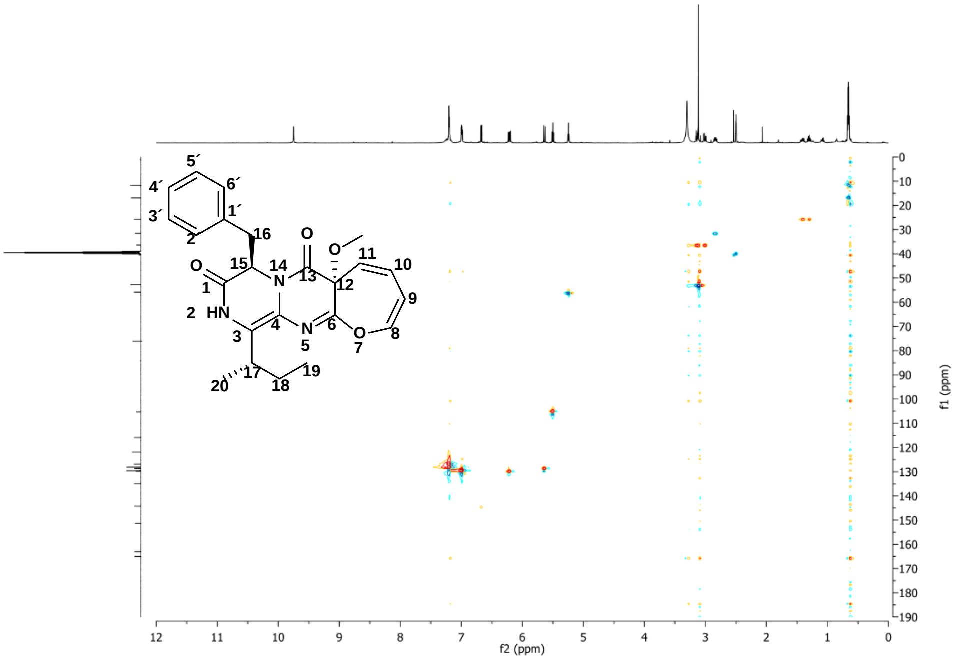

Supplementary Figure 27. HSQC spectrum of 15-*epi*-oxepinamide F (6) in DMSO-d$_6$

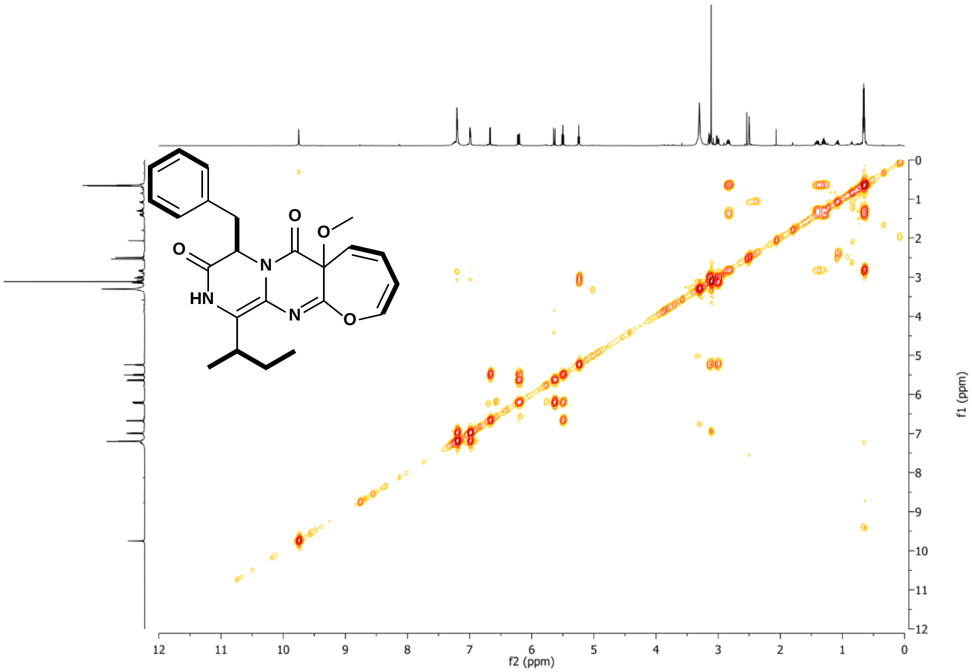

Supplementary Figure 28. COSY spectrum of 15-*epi*-oxepinamide F (6) in DMSO-d$_6$

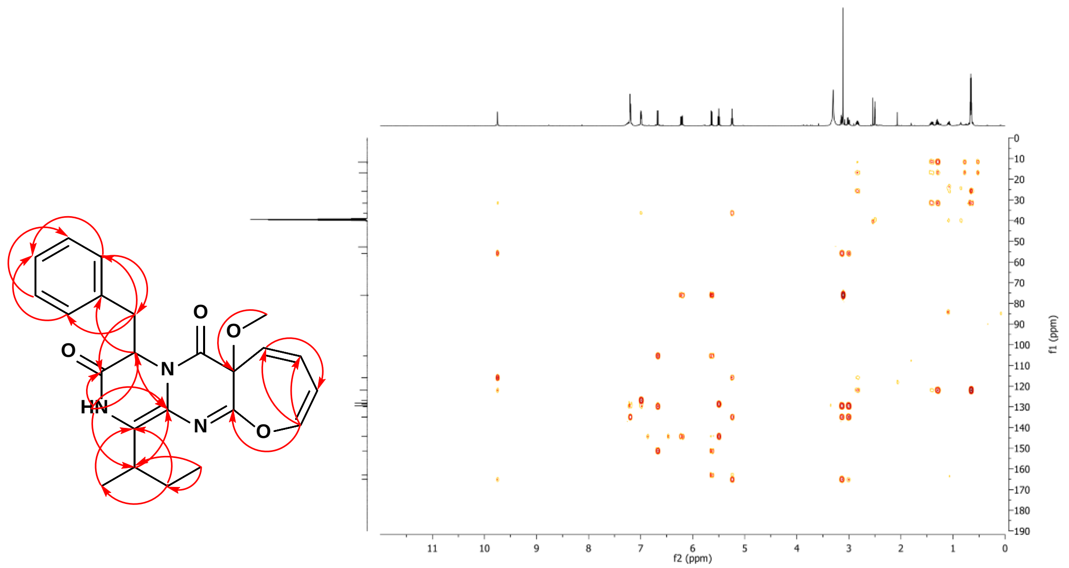

Supplementary Figure 29. HMBC spectrum of 15-*epi*-oxepinamide F (6) in DMSO-d_6

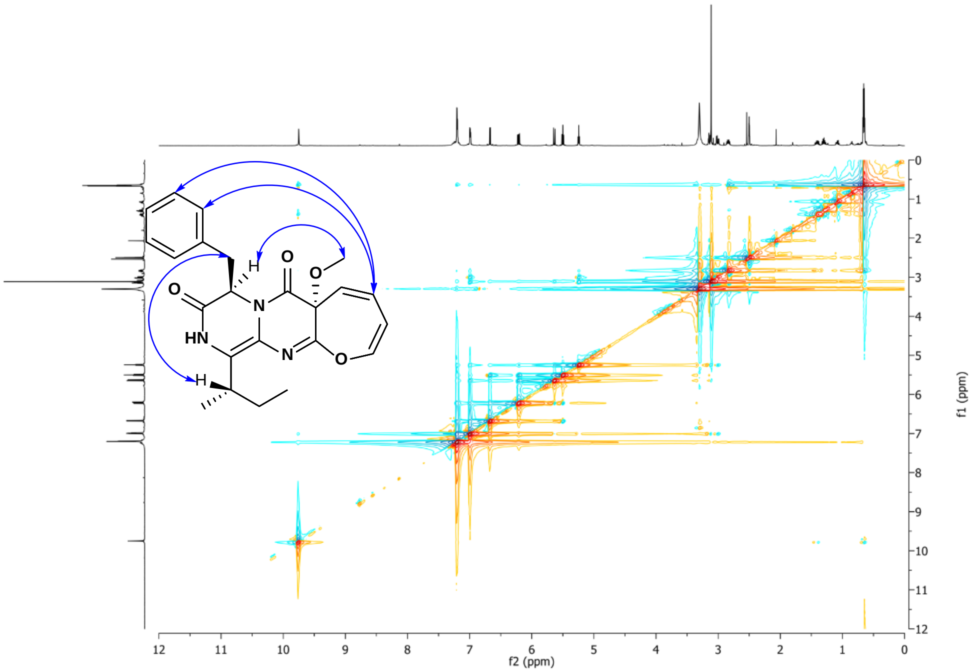

Supplementary Figure 30. NOESY spectrum of 15-*epi*-oxepinamide F (6) in DMSO-d_6

Supplementary Note 1

Physiochemical properties of the compounds described in this study.

Oxepinamide F (**1**): orange oil; $[\alpha]^{25}_{D}$ +480 (c 0.25 , $CHCl_3$) ; ^1H and ^{13}C NMR data see Supplementary Table 5; HRMS (ESI) *m/z*: $[M + H]^+$ calcd. for $C_{23}H_{25}N_3O_4$ 408.192; found 408.192 .

Oxepinamide E (**2**)**:** red brown power; $[\alpha]^{25}_{D}$ +517 (c 0.25 , $CHCl_3$) ; ^1H and ^{13}C NMR data see Supplementary Table 5; HRMS (ESI) *m/z*: $[M + H]^+$ calcd. for $C_{22}H_{23}N_3O_4$ 394.176; found 394.177.

Protuboxepin K (**3**)**:** yellow oil; $[\alpha]^{25}_{D}$ -236 (c 0.1 , $CHCl_3$) ; ^1H, ^{13}C NMR, HMBC, and NOESY data see Supplementary Table 6; HRMS (ESI) *m/z*: $[M + H]^+$ calcd. for $C_{22}H_{23}N_3O_2$ 362.186; found 362.189.

Protuboxepin A (**4**)**:** orange oil; $[\alpha]^{25}_{D}$ -362 (c 0.35 , MeOH) ; ^1H and ^{13}C NMR data see Supplementary Table 5; HRMS (ESI) *m/z*: $[M + H]^+$ calcd. for $C_{22}H_{23}N_3O_3$ 378.181; found 378.182.

15-*epi*-oxepinamide E (**5**)**:** red brown power; $[\alpha]^{25}_{D}$ +10 (c 0.23 , $CHCl_3$); ^1H, ^{13}C NMR, HMBC, and NOESY data see Supplementary Table 7; HRMS (ESI) *m/z*: $[M + H]^+$ calcd. for $C_{22}H_{23}N_3O_4$ 394.176; found 394.176.

15-*epi*-oxepinamide F (**6**)**:** red brown power; $[\alpha]^{25}_{D}$ +10 (c 0.15 , $CHCl_3$); ^1H, ^{13}C NMR, HMBC, and NOESY data see Supplementary Table 8; HRMS (ESI) *m/z*: $[M + Na]^+$ calcd. for $C_{23}H_{25}N_3O_4$ 408.192; found 408.192.

Structural elucidation.

The structures of oxepinamide F (**1**)**,** E (**2**)**,** protuboxepin K (**3**), and protuboxepin A (**4**) were elucidated by comprehensive interpretation of their MS, optical rotation, and NMR data (Supplementary Table 5, Supplementary Table 6, Supplementary Figures 7-18) and comparison with those reported in the literature.[1-3]

15-*epi*-oxepinamide E (**5**) shares the same $[M + H]^+$ ion and similar NMR data with oxepinamide E (**2**) (Supplementary Table 7, Supplementary Figures 19-24), but exhibit different cotton effects in their CD spectra. Correlation of H-17 to H-16 in NOESY spectrum of **5** proved the 15*S* configuration.

15-*epi*-oxepinamide F (**6**) shares the same $[M + H]^+$ ion and similar NMR data with oxepinamide F (**1**). Correlation of H-17 to H-16 and 12-OCH_3 to H15 in NOESY spectrum of **6** confirmed the15*S* configuration.

Supplementary References

1. Lu,X. *et al.* Oxepinamides: Novel liver X receptor agonists from *Aspergillus puniceus*. *Eur. J. Org. Chem.* **2011**, 802-807 (2011).
2. Lee,S.U. *et al.* Protuboxepins A and B and protubonines A and B from the marine-derived fungus *Aspergillus* sp. SF-5044. *J. Nat. Prod.* **74**, 1284-1287 (2011).
3. Ohte,S. *et al.* A new diketopiperazine-like inhibitor of bone morphogenetic protein-induced osteoblastic differentiation produced by marine-derived *Aspergillus* sp. BFM-0085. *J. Antibiot.* **73**, 554-558 (2020).
